# Considerations on static pressure gradients in closed circulatory systems

**DOI:** 10.14814/phy2.15983

**Published:** 2024-04-11

**Authors:** Nato Popara, Denis Cvitković, Marinko Vilić, Selim Pašić

**Affiliations:** ^1^ Deparment of Physics, Faculty of Veterinary Medicine University of Zagreb Zagreb Croatia; ^2^ Deparment of Veterinary Economics and Epidemiology, Faculty of Veterinary Medicine University of Zagreb Zagreb Croatia; ^3^ Department of Physiology and Radiobiology, Faculty of Veterinary Medicine University of Zagreb Zagreb Croatia

**Keywords:** blood circulation, siphon principle, static pressure, vascular waterfall

## Abstract

Siphons are devices that transport liquids uphill between two containers. It has been proposed that a siphon principle operates in closed circulatory systems, as best exemplified by the circulation of blood in mammals. This principle is supposed to ensure that no additional work is necessary to pump blood above the level of the heart, and that there is no gravitational static pressure gradient in the column of blood. The first statement is correct, while we demonstrate that, ignoring hydraulic resistance to blood flow, the static pressure gradient is equal to the hydrostatic gradient in a siphon model of blood circulation, although the details of the proof do not depend on the geometry of the circulatory system and the proof can be trivially extended to other models such as a vascular waterfall. This implies that the controversy over the siphon principle has no implications for the description of blood circulation, and that mechanisms such as the “baffle,” which some authors have appealed to in order to obtain the expected gradient, are not necessary. In our discussion, we also discuss empirical data that appear to provide additional verification of our results, as well as several everyday occurrences that provide additional support.

## 1 INTRODUCTION

A siphon is a device, in the most straightforward implementation a tube of constant cross‐section bent into an inverted U‐shape, with sides of unequal length (for real and ideal fluids) or equal length (for ideal fluids only), immersed into a reservoir of liquid at both ends, which transports liquids against gravity (“uphill”) without the use of a pump. Siphons have attracted scientific attention mostly due to a disagreement about the nature of the uphill flow of liquid and whether this flow is due to atmospheric pressure, gravity, or both (Stephen, 2011).

In the field of physiology, however, another “siphon controversy” has developed over a proposed “siphon principle” which equates the circulation of blood in mammals in a closed loop (such as that formed between the heart and the brain) with the operation of a siphon (Badeer, 1988, 1997; Badeer & Hicks, 1992; Gisolf et al., 2005; Hicks & Munis, 2005; Hughes et al., 2006; van der Walt et al., 2022). This is claimed to have two effects: first, that the heart does not need to work against gravity to pump blood uphill, for example through the blood vessels in the neck of a long‐necked mammal (a textbook example is the giraffe). Second, the gravitational pressure gradient uphill is supposed to differ from the gradient arising from hydrostatic pressure:

(1) $${\text{∇}}_{z}P_{\text{grav}}=-\mathrm{\rho g},$$

where *z* is height in the column of blood (in the direction of gravity). In this work, we use the gradient in its usual sense, that is, the gradient is a vector operator oriented along the greatest change of a scalar function:

(2) $$\vec{\nabla }=\frac{\partial }{\partial x}\vec{i}+\frac{\partial }{\partial y}\vec{j}+\frac{\partial }{\partial z}\vec{k},$$

where $\vec{i}$, $\vec{j}$, and $\vec{k}$ are the unit vectors along, respectively, the *x*, *y*, and *z* coordinate axes. In terms of finite differences, Equation (1) can be written as (in accordance with the definition of a gradient, we only consider the change in the variable *z*, while all other variables are frozen including time *t* from this point onwards):

(3) $$\Delta P_{\text{grav}}={\text{∇}}_{z}P_{\text{grav}}\;\Delta z=-\mathrm{\rho g}\Delta z.$$

This claim about the pressure gradient is rarely stated explicitly, but clearly follows from claims of a pressure gradient of the opposite sign and one tenth of the value predicted by the expression above in the jugular vein of a standing giraffe (as evidence of a “siphon principle” operating) (Hicks & Munis, 2005), as well as related discussions about distance from the heart to the head in various animals (Hicks & Munis, 2005) and an alternative explanation for the high aortic blood pressure in a giraffe (Badeer, 1997). A more explicit statement that the gravitational pressure of arterial blood flow is “counterbalanced” by the gravitational pressure of venous blood flow is given in Badeer (1997). A clear statement that, in the case of a “siphon principle,” operating blood pressure is invariant with respect to body size (i.e., the distance between the heart and the brain) is given in White and Seymour (2014).

In evaluating claims about a siphon principle operating in the circulation of blood, we also need to distinguish between two claims: first, that, if viscous forces are negligible, the energy expended to raise an infinitesimal volume of blood in the ascending branch of the circulatory system is compensated by an increase in the energy in the descending branch such that the overall work integral is zero. Second, that the work integral is zero by parts, in either branch of the circulatory system. The first claim is, in fact, trivially true for any conservative force, which gravity is. It does not follow that no energy is necessary to moving blood uphill. The second claim is much stronger and does imply that no energy is expended on the uphill movement of blood. In fact, the second claim is true. However, it only means that the work integral is piecewise zero. It should not be taken to mean there is no pressure gradient in the column of blood, although such a gradient is sometimes explained as the consequence of the heart having to perform extra work pumping blood uphill.

In this work, we demonstrate the existence of a hydrostatic pressure gradient in a siphon model of blood circulation. This result can be trivially extended to other models such as a “vascular waterfall” (Badeer, 1988) as the specifics of the derivation do not depend on the geometry of the fluid.

## 2 METHODS

The present work is primarily an analytic one, relying on a mathematical–logical derivation from well‐established (and experimentally verified) physical laws. As such, we have no particular experimental methods or materials to report.

## 3 RESULTS

### 3.1 Pressure gradients in a siphon model of blood circulation

**FIGURE 1:**
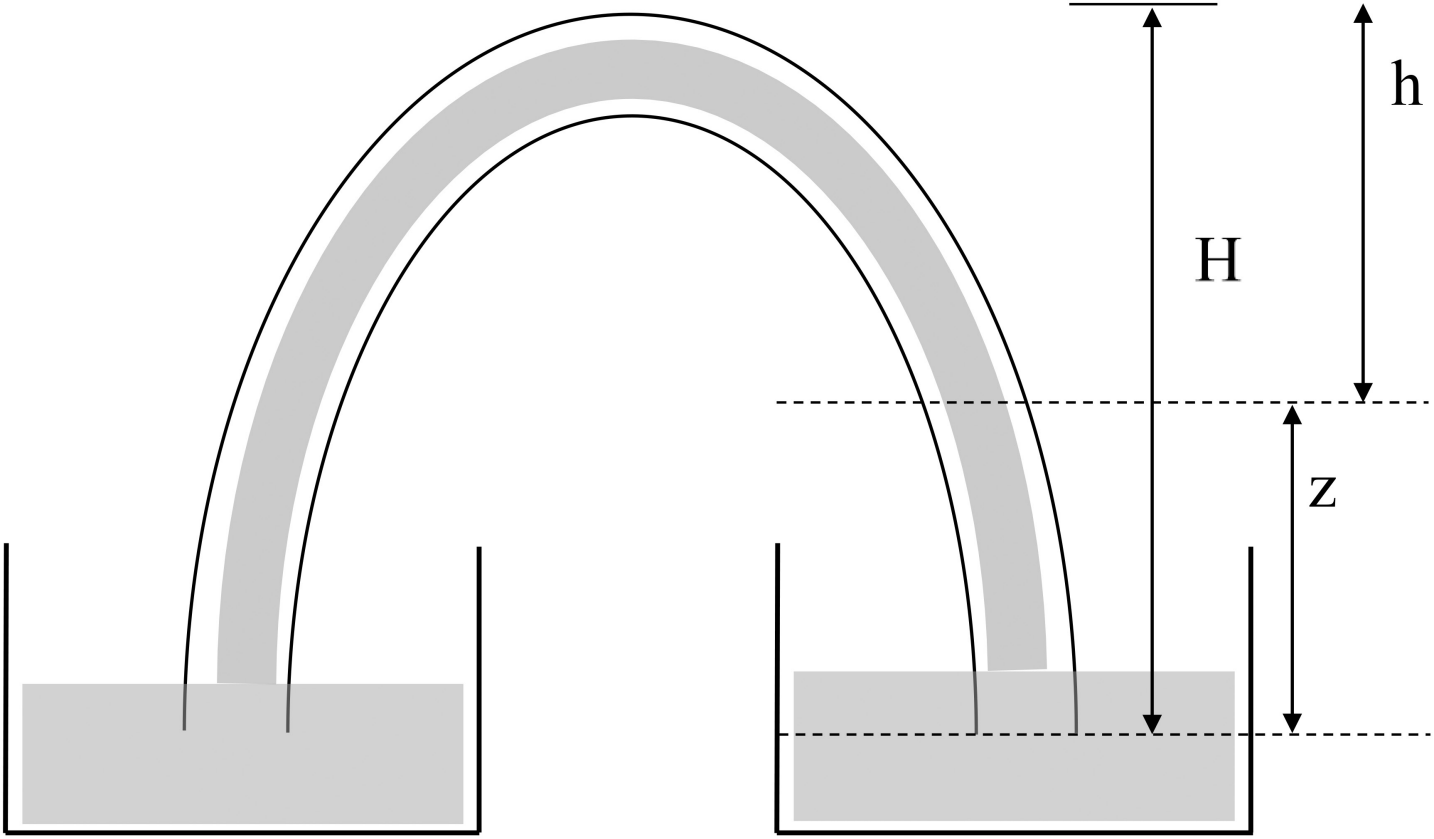
A simplified siphon with two arms of equal length. The position of an arbitrary infinitesimal volume of fluid is marked with a dotted line, and the height of the volume (denoted “*z*”), the height of the column of liquid above the position of the infinitesimal volume (*h*), and the total height of the column of liquid in the siphon (*H*) are shown.

The existence of such a gradient is easily verified, and in fact this can be done for an actual siphon. Take a simple implementation of the siphon as illustrated in Figure 1, filled with an ideal fluid (which means there is no contribution from hydraulic resistance). The sides of the siphon can be taken as being of equal length without the loss of generality. The *z* = 0 position and the position of an arbitrary point in the siphon column are also marked. Then it is easy to verify that, for any point in the column of liquid, its height *z* and the length *h* of the remaining column of fluid above the point are connected by:

(4) z+h=H,

where *H* is the overall height of the column of liquid in the siphon. We know that hydrostatic pressure is present in the system along with a constant pressure *p*_0_ (constant throughout column of liquid) due to cardiac contractions:

(5) $$P_{\text{stat}}=\mathrm{\rho gh}+p_{0}\left(t\right)$$

Taking a small finite difference of this expression, we can derive:

(6) $$\Delta P_{\text{stat}}=\mathrm{\rho g}\Delta h=\mathrm{\rho g\Delta}\left(H-z\right)=-\mathrm{\rho g}\Delta z=\Delta P_{\text{grav}},$$

since *H* and *p*_0_ are both constants in the *z* direction, whose variation in *z* direction is zero. In the end, we obtain the expected result, a negative pressure gradient with increasing height *z*, due to hydrostatic pressure.

The question naturally arises if any additional contribution to pressure might change this result. However, this is not the case, as a simple argument will demonstrate. Such an additional contribution to pressure can only come from outside the column of liquid. This happens, for example, with the negative pressure generated by the flow of liquid out of the end of a siphon. Such pressures, however, are subject to Pascal's law given the near‐incompressibility of blood (Barrett et al., 2015; Hall, 2015), and will therefore be constant throughout the column of blood. It follows, then, that they cannot represent an additional contribution to the pressure gradient.

Given the simplicity of this derivation, and the fact that it was explicitly derived for a siphon model, we might ask ourselves how claims about a pressure gradient that is different not only in value but also in sign (Hicks & Munis, 2005) became accepted among those advocating a siphon model of blood circulation. As mentioned, part of the problem is probably that the negative pressure gradient is sometimes “explained” by the work done to pump blood upstream, which would naturally mean that no such gradient exists if the work is zero. To explore this controversy further and to demonstrate that the hydrostatic contribution cannot be dismissed, we will consider an argument for the “siphon principle” in Hicks and Munis (2005).

### 3.2 Static pressure and gravitational potential energy

We will reproduce only the outlines of the argument here. In addition, while the authors start from a combination of Bernoulli's and the empirical Pouiselle equation (Synolakis & Badeer, 1989) for stationary laminar flow of a viscous fluid in narrow cylindrical vessels, the viscous forces represented by the Pouiselle equation do not contribute to the argument. Therefore, we will start with Bernoulli's equation written in terms of total energy:

(7) $$E_{\text{total}}=\left(P_{\text{stat}}+\mathrm{\rho gz}+\frac{1}{2}\rho v^{2}\right)∆V.$$

Here, the first term in the parentheses denotes static pressure, the second the gravitational potential energy per unit volume, and the third is conventionally called dynamic pressure. The static pressure contribution can be written as:

(8) $$P_{\text{stat}}=p_{0}+\mathrm{\rho gh}+P_{\text{drop}},$$

where the first term on the right‐hand side represents the static pressure due to the contractions of the heart, the second is the hydrostatic pressure that we know exists in any fluid in a gravitational field, and the last represents any further contributions to static pressure that may arise. From Equations (5) and (2), we can write:

(9) $$\begin{array}{c} E_{\text{total}}=\left(p_{0}+\mathrm{\rho g}\left(H-z\right)+\mathrm{\rho gz}+P_{\text{drop}}+\frac{1}{2}\rho v^{2}\right)∆V\\ \;=\left(p_{0}+P_{\text{drop}}+\mathrm{\rho gH}+\frac{1}{2}\rho v^{2}\right)∆V. \end{array}$$

Furthermore, we know that the total energy along a streamline is constant, and thus we can write:

(10) $$E_{\text{total}1}-E_{\text{total}2}=\left(P_{\text{drop}1}+\frac{1}{2}\rho {v_{1}}^{2}-P_{\text{drop}2}-\frac{1}{2}\rho {v_{2}}^{2}\right)∆V=0.$$

The term consisting of the dynamic pressure multiplied by a small volume is the kinetic energy. The other terms, therefore, represent potential energy. The value of the work integral for a conservative force, as is well‐known from elementary mechanics, is equal to the difference between the potential energies at the two end‐points of the curve over which the work integral is evaluated:

(11) $$W=E_{\mathrm{pot}1}-E_{\mathrm{pot}2}=P_{1}^{\prime }-P_{2}^{\prime }=\frac{1}{2}\rho \left(v_{2}^{2}-v_{1}^{2}\right)∆V.$$

We see that the only contribution to the work integral comes from the static pressure drops due to changing dynamic pressure. However, we expect the above to be true even in cases where the difference in velocities between the two points is negligible (such as a siphon with a constant cross‐section), and can in that case write with no loss of generality:

(12) $$W=E_{\mathrm{pot}1}-E_{\mathrm{pot}2}=\frac{1}{2}\rho \left(v_{2}^{2}-v_{1}^{2}\right)∆V=0,$$

and therefore, the work integral is piecewise zero (Hicks & Munis, 2005). However, as is clear from Equations (8) and (9), the hydrostatic contribution is crucial for the above derivation to be valid. Without this contribution, changes in gravitational potential energy would not be compensated with a change in hydrostatic pressure (the sum of both contributions being equal to the constant ρgH), and the potential energy would have a dependence on *z*. But as shown at the start of this section, if the hydrostatic pressure exists in the column of blood, the pressure gradient will be of the form specified in Equation (6).

Furthermore, we would like to note that the form of Equation (9) might lead to an incorrect conclusion that hydrostatic pressure is constant. This misunderstanding concerns the gravitational potential energy. It is true that the energy contributions from gravitational potential energy and hydrostatic pressure multiplied by unit volume can be added together, meaning that changing hydrostatic pressure does not result in a change in the overall potential energy as the associated change in energy is canceled out by the change in gravitational potential energy. But when we consider the problem in terms of pressure rather than energy, we need to note the following. The gravitational potential energy per unit volume (ρgz), despite having the units of pressure, is not the same kind of physical quantity as (static) pressure and hence cannot be added to the static pressure to cancel out the hydrostatic contribution. In fact, the two quantities apply to different systems, with the first describing the gravitational potential energy of a point in a mass distribution, while the latter strictly describes the pressure acting on an infinitesimal volume of liquid.

## 4 DISCUSSION

As we have shown, although the work integral for the inviscid flow of blood within a siphon model is piecewise zero, the pressure gradient uphill in the closed circulation loop of the siphon is not. Thus, the usual conclusions about the role pressure gradients play in determining the pressure imparted by the heart hold. In an attempt to retain these conclusions, some authors have appealed to a vaguely defined “baffle,” (Gisolf et al., 2005) which would act as a barrier in the circulation of blood and therefore, it is claimed, stop a siphon effect from operating. We conclude that no such barrier is necessary, and in any case, it would not affect the previous derivation, which is true for any volume of liquid regardless of whether it can circulate or not.

Empirical measurements of blood pressure in a standing, sedated giraffe (Hargens et al., 1987) have been claimed to show that the static pressure gradient differs from the one predicted by considerations of hydrostatic pressure due to the operation of a siphon in the circulation of blood (Badeer & Hicks, 1992). However, as has been noted by other authors (Badeer, 1988), these measurements did not take into account viscous resistance. Furthermore, the recent measurements of blood pressure in standing giraffes (Aalkjær & Wang, 2023; Østergaard et al., 2011) clearly show a pressure gradient of a form that would be expected from the hydrostatic contribution, providing additional empirical support to our derivation. The authors' result for the blood pressure gradient (64 ± 0.2) mmHg/m are slightly less than the hydrostatic contribution (76 mmHg/m). This is easily explained by the additional effects of hydraulic resistance of the blood flow. In addition, we suspect that the standard deviation of the slope coefficient quoted in the article should be significantly higher due to the fact that measurements were performed on animals with significantly varying heights (important because the height was measured from the ground). The measurements discussed were not specifically intended to obtain the hydrostatic pressure gradient, meaning that there is room for a more direct experimental verification. Arguments from the scaling of systolic pressure with body mass as presented in Badeer and Hicks (1992) and van der Walt et al. (2022) are not convincing, first because body mass is a poor proxy for mean head‐heart height when considering animals with diverse anatomies including long‐necked mammals such as giraffes, and second because the hydrostatic pressure gradient only provides a lower bound for systolic pressure. Other contributions can lead to higher systolic pressures.

Our results are valid not only regardless of the model but also regardless of the kind of animal as long as the animal has a closed circulatory system. The debate about the siphon mechanism has mostly concerned terrestrial mammals, although birds (Badeer & Hicks, 1992) and sauropod dinosaurs (Hughes et al., 2006) have also been considered, but our results apply to, for example, aquatic mammals such as whales as well (regardless of the buoyancy force, which acts in their center of gravity, not along the column of blood).

We would also like to point out several everyday occurrences which also lend support to the existence of a hydrostatic pressure gradient in the circulation of blood. One such occurrence is the dizziness or even loss of consciousness that may accompany sudden changes in the height of the head, such as when a person gets up very rapidly. Another is the fact that patients with cuts on their hands are encouraged to raise them to slow down the flow of blood (due to a significantly lower blood pressure when the blood vessels at the position of the cut do not experience the additional hydrostatic contribution). We may also note the rapid onset of fatigue that accompanies work done with hands extended above the head, again due to a significantly lower blood pressure.

Finally, given that the work integral for an infinitesimal volume of fluid moving in the column of blood is piecewise zero, we might ask if this means anything for the work that the heart has to perform to pump blood. In fact, it means little, as the work that the heart needs to perform, as in any circular pump, is given by the product of the static pressure at the point where the heart pumps blood and the stroke volume (Stepanoff, 1993). Therefore, a long‐necked animal will need to maintain a higher blood pressure to avoid a significant negative pressure in the head when the neck is vertical, meaning a higher work for the heart. This work is not expended to move blood but is done against friction forces, in blood and in the muscle fibers of the heart itself.

## FUNDING INFORMATION

No funding information provided.

## CONFLICT OF INTEREST STATEMENT

No conflicts of interest, financial or otherwise, are declared by the authors.

## ETHICS STATEMENT

Given that the research presented in this article did not involve human or animal experimentation, no particular ethics statement is necessary.

## Data Availability

The data that support the findings of this study are available from the corresponding author upon reasonable request.
